# Supplementary material for: The Yellow Stripe-Like (YSL) Gene Functions in Internal Copper Transport in Peanut
Source: Genes (Basel). 2018 Dec 14;9(12):635. doi: 10.3390/genes9120635 (PMC6316571; doi:10.3390/genes9120635)
Supplement: Supplementary file 1 [file genes-09-00635-s001.pdf]

genes

MDPI

Supplementary data

Supplementary Fig. S1

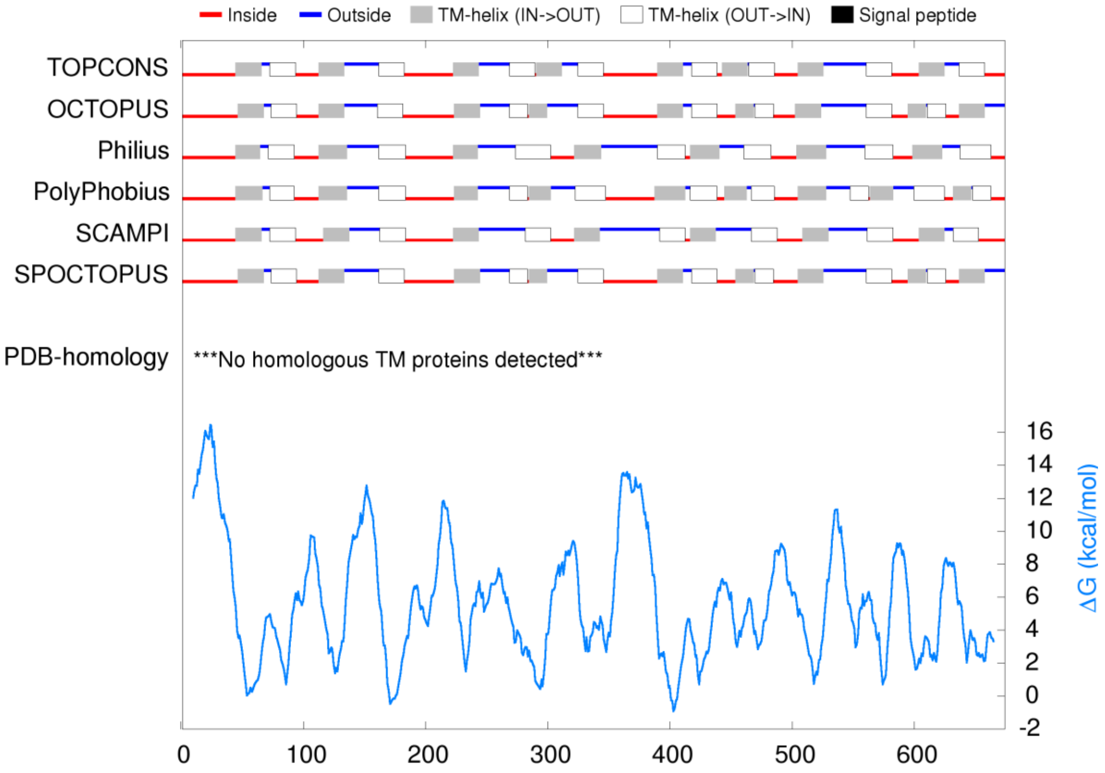

Supplementary Fig. S1 The predicted membrane protein topology and signal peptide of AhYSL3.1 based on different methods.

Supplementary Fig. S2

A

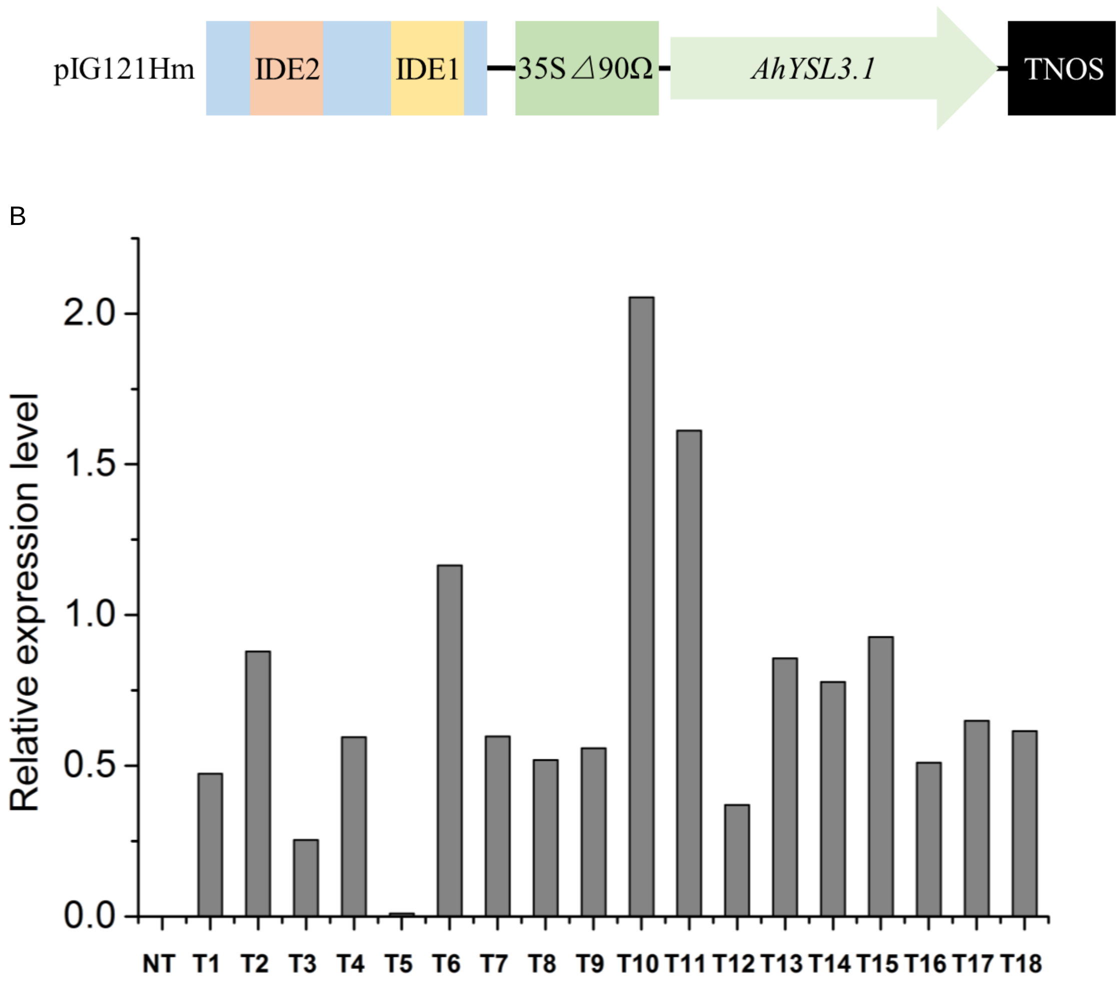

Supplementary Fig. S2 Generation of *AhYSL3.1*-induced transgenic plants. (A) Schematic diagram of the binary vector for induction of AhIRT1 expression. Expression of the AhYSL3.1 gene was driven by an artificial promoter containing IDE1 and IDE2, which are cis-acting elements conferring Fe-deficiency-specific expression in tobacco roots, fused to the -90/+8 region of the cauliflower mosaic virus 35S promoter and a 5’ leader (Ω) sequence of the tobacco mosaic virus to enhance basal expression (Kobayashi et al., 2003; Kobayashi et al., 2004). The pIG121Hm vector was used as a backbone. TNOS is the terminator of the nopaline synthase gene. (B) *AhYSL3.1* expression levels in shoots of the NT and transgenic lines. NT, non-transformed plants; T1–T18, transgenic lines.

Supplementary Fig. S3

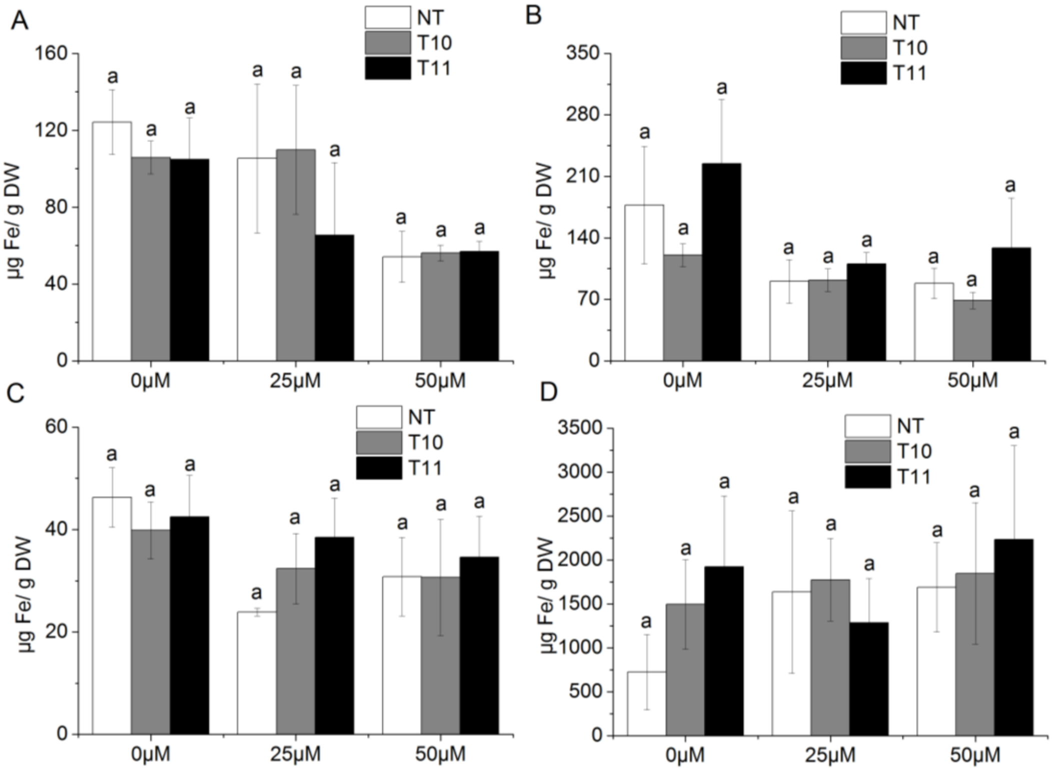

Supplementary Fig. S3 Fe concentrations in the NT and transgenic lines treated with various concentrations of Cu. (A) Young leaves, (B) old leaves, (C) stems, and (D) roots.

Supplementary Fig. S4

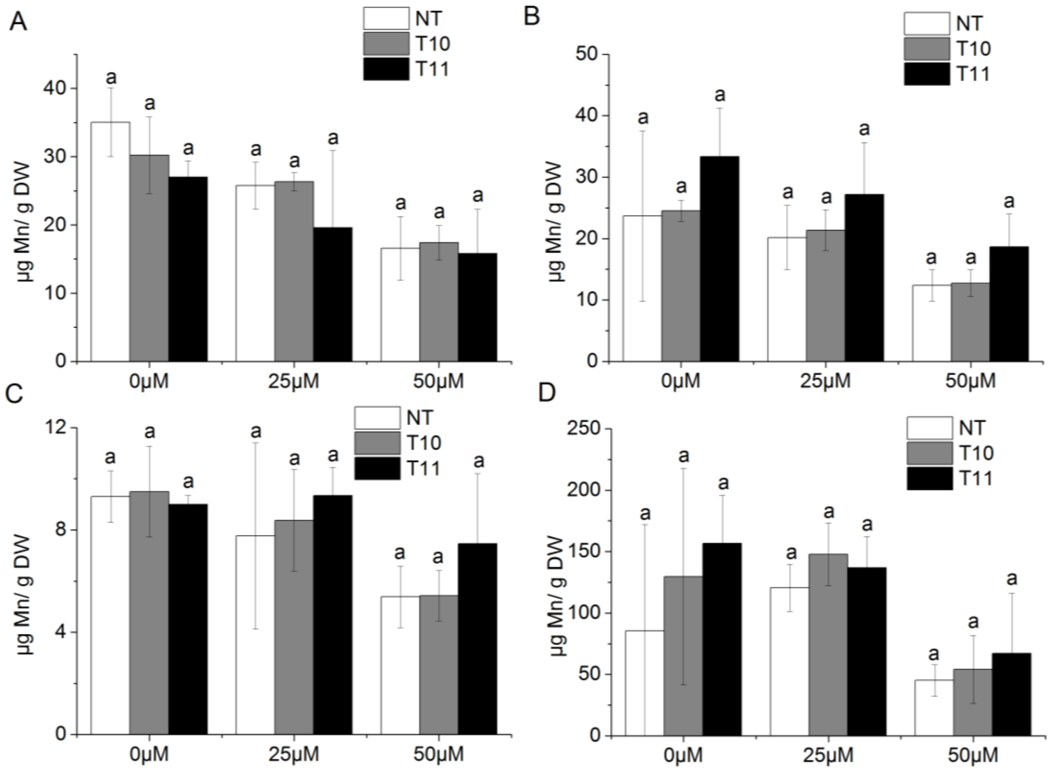

Supplementary Fig. S4 Mn concentrations in the NT and transgenic lines treated with various concentrations of Cu. (A) Young leaves, (B) old leaves, (C) stems, and (D) roots.

Supplementary Fig. S5

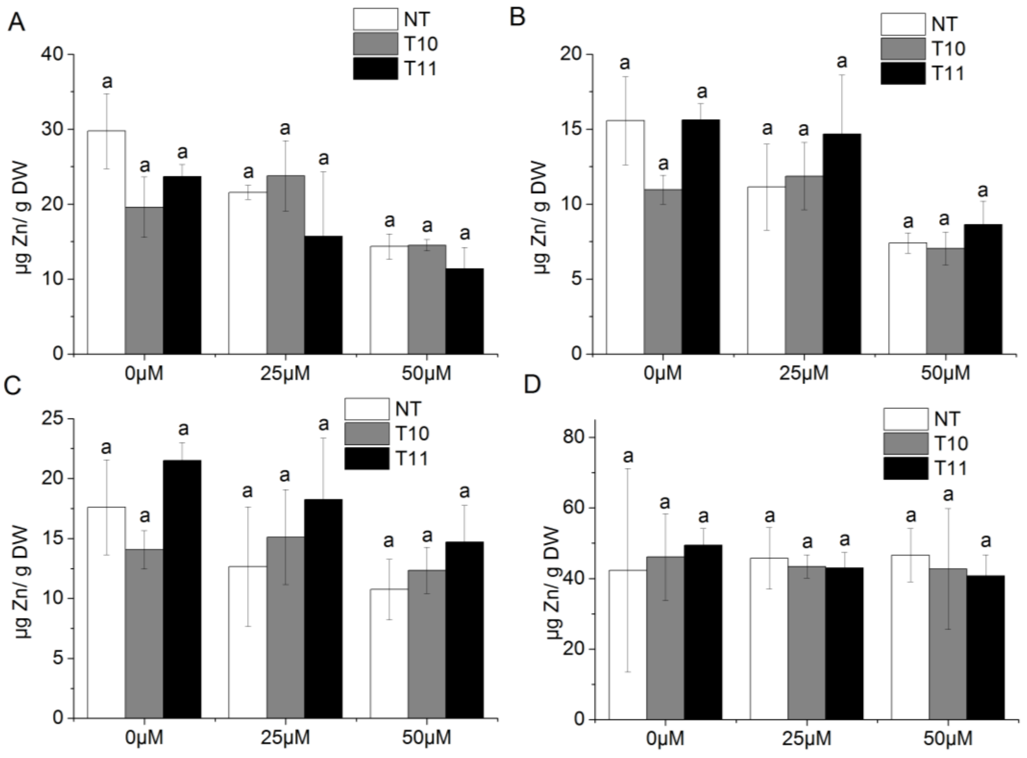

Supplementary Fig. S5 Zn concentrations in the NT and transgenic lines treated with various concentrations of Cu. (A) Young leaves, (B) old leaves, (C) stems, and (D) roots.

Supplementary Fig. S6

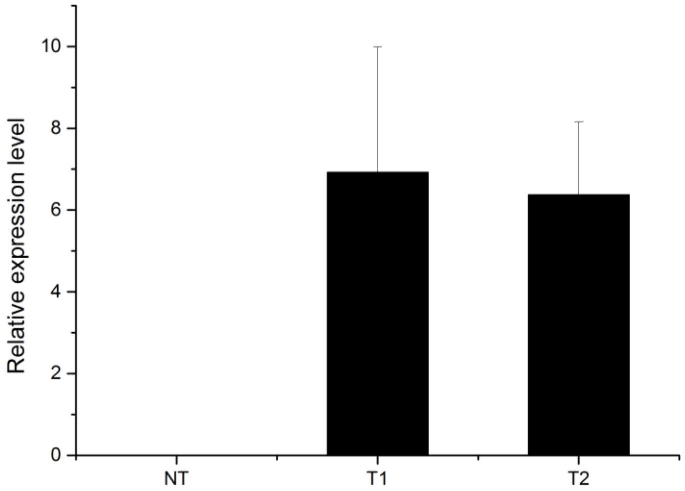

Supplementary Fig. S6 Relative expression level of *AhYSL3.1* in young leaves of NT and transgenic rice plants under excess Cu conditions.

Supplementary Fig. S7

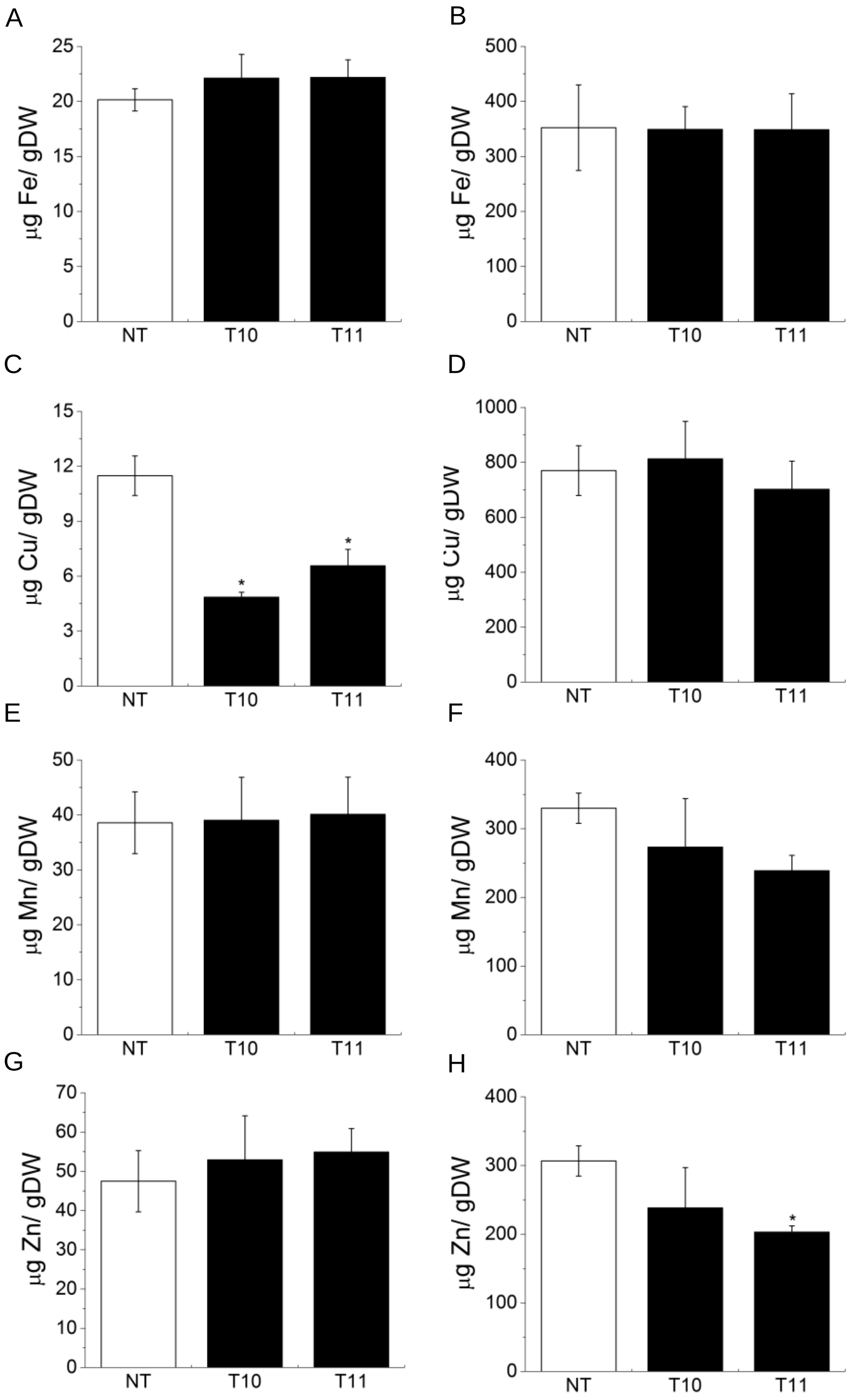

Supplementary Fig. S7 Metal concentrations in young leaves and roots of transgenic and NT tobacco plants under Fe-deficient conditions. The tobacco plants were Fe-deprived for 9 days. (A, B) Fe, (C, D) Cu, (E, F) Mn, and (G, H) Zn concentrations in new leaves (A, C, E, G) and roots (B, D, F, H) of NT and transgenic plants. Results are presented as means ± SD of triplicate samples. Significant differences from NT were determined by Student's *t*-test, **P* < 0.05.

Supplementary Fig. S8

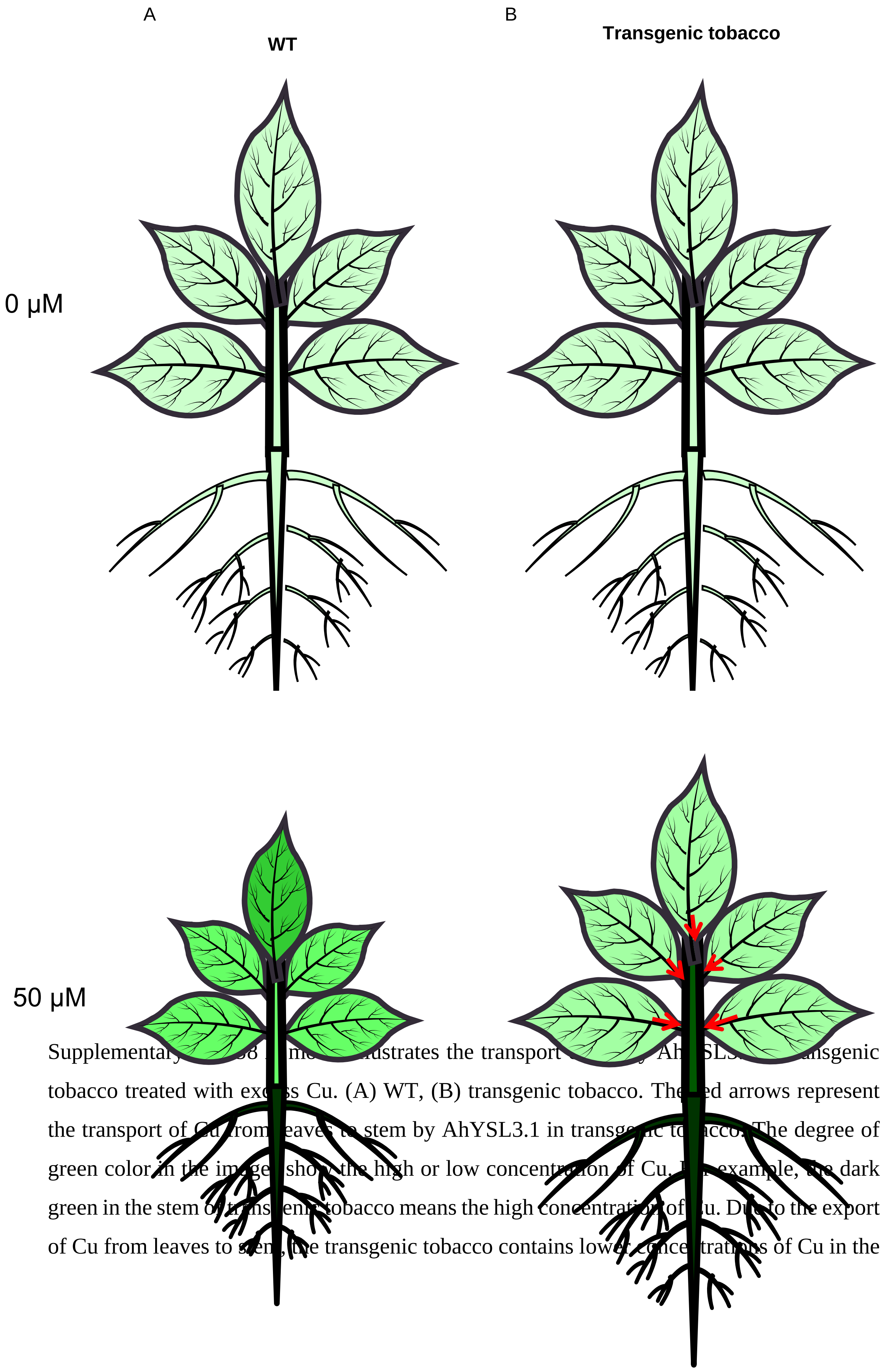

Supplementar[illegible] S8 [illegible]me [illegible]llustrates the transport [illegible] Ah[illegible]SL3.[illegible]ansgenic tobacco treated with excess Cu. (A) WT, (B) transgenic tobacco. The [illegible]ed arrows represent the transport of Cu from leaves to stem by AhYSL3.1 in transgenic tobacco. The degree of green color in the images show the high or low concentration of Cu. F[illegible] example, the dark green in the stem of transgenic tobacco means the high concentration of Cu. Due to the export of Cu from leaves to stem, the transgenic tobacco contains lower concentrations of Cu in the

leaves and higher concentrations of Cu in the stem compared to the WT in the excess Cu condition. Thus, the transgenic plants are tolerant to excess Cu.
